# Supplementary material for: The genome sequence of a tephritid fruit fly, Xyphosia miliaria (Schrank, 1781) (Diptera: Tephritidae)
Source: Wellcome Open Res. 2025 Oct 21;10:585. [Version 1] doi: 10.12688/wellcomeopenres.25047.1 (PMC12749564; doi:10.12688/wellcomeopenres.25047.1)
Supplement: Peer review history

# Reviewer report: Reviewer response for version 1

**DOI:** 10.21956/wellcomeopenres.27613.r140485

**Published:** 2025-12-29

**Authors:** Matsapume Detcharoen

**Affiliations:** 1 Division of Biological Science, Faculty of Science, Prince of Songkla University, Hat Yai, Songkhla, Thailand, Hat Yai, Thailand

The paper presents a high quality, phased reference genome for the tephritid fruit fly *Xyphosia miliaria*. The authors assembled two haplotypes of roughly 807 Mb and 800 Mb, with most of haplotype 1 assigned to seven chromosome scale scaffolds that include the X and Y chromosomes, and most of haplotype 2 assigned to five autosomal chromosomes. Assembly quality is very high and 98.6 percent complete BUSCO. The authors highlight that this resource will support studies of fly phylogeny, host plant use, parasitoid interactions, and broader biodiversity genomics efforts.

Are sufficient details of methods and materials provided to allow replication by others?

Yes

Is the rationale for creating the dataset(s) clearly described?

Yes

Are the datasets clearly presented in a useable and accessible format?

Yes

Are the protocols appropriate and is the work technically sound?

Yes

Reviewer Expertise:

Molecular ecology

I confirm that I have read this submission and believe that I have an appropriate level of expertise to confirm that it is of an acceptable scientific standard.

# Reviewer report: Reviewer response for version 1

**DOI:** 10.21956/wellcomeopenres.27613.r140480

**Published:** 2025-11-25

**Authors:** Daubian Santos

**Affiliations:** 1 Universidade Federal do ABC, Santo André, State of São Paulo, Brazil

The article is very good and a part of the excellent  Darwin Tree of Life Project.

 Good writing and background well stablished. The methodology is also clear and efficient.

 I only said two small comments:

 - Tephritidae is the largest of the picture-winged fly families in general, not only in Britain.

 - Although it is not obligated, the authors may include the reference of species authors original descriptions cited in the text (for example, Robineau-Desvoidy, 1830) in the references.

Are sufficient details of methods and materials provided to allow replication by others?

Yes

Is the rationale for creating the dataset(s) clearly described?

Yes

Are the datasets clearly presented in a useable and accessible format?

Yes

Are the protocols appropriate and is the work technically sound?

Yes

Reviewer Expertise:

Taxonomy, systematics, protocols of extracting genetic data

I confirm that I have read this submission and believe that I have an appropriate level of expertise to confirm that it is of an acceptable scientific standard.
